# Quo Vadis?

**Published:** 1902-07

**Authors:** 


### Quo Vadis?

SINCE the first leader in this edition was written we have observed, by the reported proceedings of the house of delegates at Saratoga, that a proposed code of ethics was introduced in that body and referred to a committee instructed "to examine and report for final action" next year.

In this document, Section 2 of Article IV. reads: "The good of the patient being the sole object in view, any physician having a license to practise medicine conferred by the state may be aided in consultation." The next section (3) of the same article reads: "No physician who indicates to the public that his practice is based on a sectarian system of medicine shall be entitled to professional fellowship or to recognition in medical bodies." There seems to be reason for comment upon this extraordinary instrument at this time, especially upon these two remarkable sections, so contradictory in phraseology and so lacking in that affability which is in keeping with the spirit of harmony that everywhere prevails.

In the first place it should be remembered that the American Medical Association recently evolved from the academic to the incorporate state. In its former condition it made much of its code of ethics, a form of words entirely appropriate to the period of its adoption, but which served for half a century to repress independence in medicine. As an academic body, corresponding as it did to a large debating society or lyceum, it could adopt a code suited unto itself, no matter how intolerant or contrary to law, and yet be consistent and logical; but when it became an incorporate body with a legal existence, a code in any degree contrary to the provisions of the laws of any state became inoperative because illegal.

It may be observed of the first section above quoted that it is of questionable propriety, because an assumption of superiority is not good form in any walk of life, and is almost sure to be resented by those whom it concerns the most. As to the next section, also quoted, it is clearly contrary to the law of every state or political division having a state examining board, now some fifty in number; besides, its enactment would be contrary to the terms of the statute under which the association is incorporated, hence void of effect.

There are no sects in medicine known to law, and to refer to sectarian physicians in specific terms is quite out of place in this age. Moreover, those referred to as practising a "sectarian system of medicine," have been duly licensed by the state to all the rights, privileges, distinctions, duties and emoluments relating to the practice of medicine and surgery, and the courts will not hesitate to maintain them in such rights.

If the American Medical Association must needs have a code of ethics, though it seems to have run along very well for a year without one, would it not be well to make a simple, brief, dignified declaration of principles, rather than enact this archaic paper, so suggestive of the Devonian period?

These observations are based on the assumption that the purpose of the American Medical Association is in very fact what its constitution declares it to be, "to federate into one compact organisation the medical profession of the United States." This high and noble object and intention declared by the constitution, expounded by President Reed and reaffirmed by President Wyeth, cannot but mean to include every legally qualified physician in the national domain, many of whom, of course, are not now members of this great organisation. We have written seemingly in behalf of these latter, but in deeper reality on behalf of the "one compact organisation of the medical profession" which we are desirous that the American Medical Association shall become.

